# UK policy statements on testosterone deficiency

**DOI:** 10.1111/ijcp.12901

**Published:** 2017-03-20

**Authors:** Geoffrey Hackett, Michael Kirby, David Edwards, T. Hugh Jones, Jonathan Rees, Asif Muneer

**Affiliations:** ^1^ Good Hope Hospital Birmingham UK; ^2^ University of Bedfordshire Luton UK; ^3^ Faculty of Health & Human Sciences University of Hertfordshire Hatfield UK; ^4^ The Prostate Centre London UK; ^5^ White House Surgery Chipping Norton UK; ^6^ British Society for Sexual Medicine (BSSM) Staffordshire UK; ^7^ Barnsley Hospital Barnsley UK; ^8^ University of Sheffield Medical School Sheffield UK; ^9^ Royal Hallamshire Hospital Sheffield UK; ^10^ Backwell & Nailsea Medical Group Bristol UK; ^11^ Primary Care Urology Society London UK; ^12^ Department of Urology and NIHR Biomedical Research Centre University College London Hospitals London UK

## Abstract

To address widespread media and scientific concerns over the appropriate treatment of TDS with Testosterone Therapy (T Therapy), the Executive Committee of the British Society for Sexual Medicine developed eight consensus statements, based on current scientific evidence to address these controversial issues. These statements were in no‐way designed to replace the published evidence‐based guidelines on the subject developed by various professional organisations, but to provide specific answers to several current controversial issues. This review examined evidence from Medline, EMBASE and Cochrane searches on HG, T Therapy and cardiovascular safety from May 2005 to May 2015, which revealed 1714 articles, with 52 clinical trials and 32 placebo‐controlled randomised controlled trials. The task force developed the following eight key statements.

**What's known**

There have been recent controversies on the use of exogenous testosterone in men with late‐onset hypogonadism. This is a medical issue that has long been neglected and which carries both physiological and psychological complications.

**What's new**

These statements are developed for UK practice and take into account the outcomes from an International expert consensus conference on testosterone deficiency and its treatment held in Prague 2015. The statements have been developed to address widespread media and scientific concerns over the appropriate treatment of TDS with T Therapy.

## 1 Introduction

In response to recent controversies,^1^ a British Society for Sexual Medicine (BSSM) task force met to develop a consensus on the use of exogenous testosterone in men with late‐onset hypogonadism (HG). This is a medical issue that has long been neglected and which carries both physiological and psychological complications. Recent media coverage of two high profile but flawed publications has confused the situation concerning the safety of testosterone therapy (T Therapy).^2^, ^3^ In response to US pressure groups such as Public Citizen, a recent Food and Drug Administration publication^4^ highlighted the need for well‐conducted studies to clarify the risk/benefit issues. No such concerns were expressed by the European Medicine Agency.^5^

## 2 Terminology

The committee acknowledged that current international terminology caused great confusion. We have attempted to use terms consistently to promote clarity. The US preference is for the term “T Therapy” and “low T” but the committee preferred to older term “T Therapy,” which suggests a physiological replacement following measured evidence of deficiency. The older term HG is still preferred by traditional urologists and endocrinologists and is often the only term accepted by traditional medical journals. The term “late‐onset HG” is often used to describe the condition of mixed primary and secondary HG usually found in older men and is used in an attempt to differentiate from “classical” HG associated with underproduction of testosterone from traditional disorders of the testis (primary) or pituitary (secondary).

### 2.1 Testosterone deficiency is a well‐established, significant medical condition

**Figure 1:**
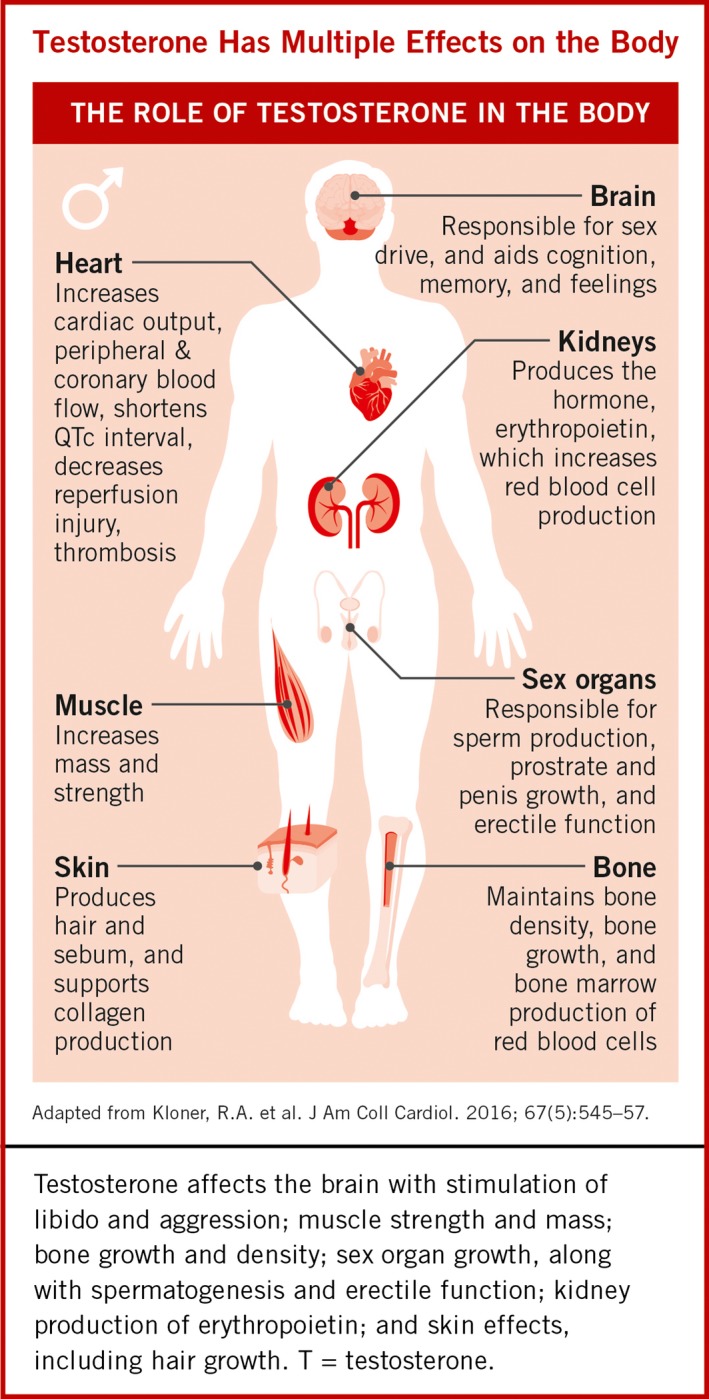
Effect of testosterone on multiple organ systems^10^

The current International Society for Study of the Ageing Male (ISSAM),^6^ European Association of Urology (EAU),^7^ International Society for Sexual Medicine (ISSM),^8^ and BSSM^9^ definition of HG or Testosterone Deficiency Syndrome (TDS) is as follows:

A biochemical syndrome associated with advancing age and characterised by a deficiency in serum androgen levels with or without a decreased genomic sensitivity to androgens. It may result in significant alterations in the quality of life and adversely affect the function of multiple organ systems (Figure 1).^10^

It is worthy of note that the ISSM,^8^ EAU^7^ and ISSAM^6^ guidelines have been updated (2015), but the Endocrine Society (ES) guidelines date from 2010^11^ and therefore do not reflect the vast literature of the amount of literature published in the last 5 years (Table 1).

**Table 1:** Current guidelines on hypogonadism

Organisation	Recommendation	TT levels	Follow‐up	Monitoring
ISSM 2015^8^	Symptomatic HG, ED and low desire	<8 nmol/L is likely to benefit8‐12 nmol/L, Check FT. Consider 6‐month trial of therapy if symptoms troublesome and continue if substantial benefit. Prolactin if TT below 5.2 nmol/L, TT>12 nmol/L unlikely to benefit	3‐6 months then annually	Baseline DRE, PSA 1.4 ng/dL, rise in any year or 0.4 per year velocity. Haematocrit 54%. Aim at T level above 15 nmol/L
BSSM 2010^9^				
EAU 2015^6^	Decreased muscle mass or BMD Decreased libido or erection	<8 nmol/L or 8‐12 nmol/L check FTConsider 6‐month trial of therapy	3‐6 months then annually	Baseline DRE, PSA 1.4ng/dl riseBaseline assessed 6 months after commencement. Haematocrit 54%. Aim at T level above 15 nmol/L
Endocrine Society 2010^7^	Symptomatic HG with unequivocal low T. Low desire and ED. High‐risk groups identified, but screening not recommended	200‐250 ng/dL=Frank hypogonadism. Prolactin if TT below 5.2 nmol/L	3‐6 months then annually	Baseline DRE. DRE plus PSA 3‐6 months, PSA 1.4 nmol/L, haematocrit 54%. Aim at T level 400‐700 ng/dL
ISSAM, ISA 2015^11^	Symptomatic HG, ED and low desire	<8 nmol/L, 8‐12 nmol/L, FT <225 pmol/L, consider 3‐ to 6‐month trial of therapy	3‐6 months then annually	Baseline DRE. DRE plus PSA 3‐6 months, PSA 1.4 nmol/L, Haematocrit 52.55%

The most relevant clinical symptoms/signs of HG, as per EAU and ISSM guidelines, are listed in Table 2.^7^, ^8^

**Table 2:** Most relevant clinical symptoms/signs of hypogonadism 7, 8

Symptoms of TD	Loss of libido
	Absence of morning and night time erections
	Erectile dysfunction
	Ejaculatory dysfunction
	Fatigue
	Reduced well‐being
	Depression
	Loss of concentration
	Hot flushes
	Reduced muscle mass and weakness
	Reduced body hair

European Association of Urology, ISSM and BSSM guidelines suggest that a level of total testosterone (TT) of <8 nmol/L or free testosterone (FT) of <180 pmol/L (based on two separate 8‐11 am levels) requires T Therapy and TT of >12 nmol/L or FT of >225 pmol/L does not. Between these levels, a trial of therapy for a minimum of 6 months should be considered based on symptoms.^7^ The ES in 2010 recommended testosterone assessment in a number of high‐risk groups, including those with type 2 diabetes mellitus (T2DM) and metabolic syndrome, along with chronic illnesses such as heart failure, renal failure, and human immunodeficiency virus (HIV), and men taking long‐term opiate analgesics and anticonvulsants.^11^ The ES advised measuring testosterone for erectile dysfunction (ED) and symptomatic HG but stopped short of recommending screening for testosterone in diabetes despite a 40% prevalence.^12^ ED and TDS have been shown in studies to be independently associated with reduced quality of life.^13^

The European Male Aging Study (EMAS) evaluated over 3000 men aged 40‐70^14^ according to biochemistry and symptoms and showed that 75% of men maintain normal testosterone levels into old age, suggesting that HG was not merely a function of ageing. The prevalence of secondary HG was 11.8%, with 2% suffering primary HG and 9.5% suffering from compensated HG worthy of observation but not T Therapy.

### 2.2 Testosterone deficiency has well‐established symptoms

The most prevalent symptoms of male HG in ageing men are reduced sexual desire and sexual activity, ED, loss of morning erections and hot flushes.^7^ Other factors found associated with low testosterone include increased waist circumference, obesity, metabolic syndrome and impaired health status. Mulligan et al. reported the odds ratio for HG with comorbid conditions in a primary care population as hypertension 1.84, dyslipidaemia 1.47, type 2 diabetes 2.09, obesity 2.38, LUTS/BPH 1.20 and COPD 1.40.^15^ Severe HG is associated with increased risk of osteoporosis and chronic anaemia.

Other less‐specific symptoms are loss of physical strength and muscle mass, fatigue, changes in mood, anger, sleep disturbance and cognitive impairment. Classical signs are decreased body hair, gynaecomastia, and decreased testicular volume. Signs and symptoms of androgen deficiency vary depending on age of onset, duration and the severity of the deficiency.^7^ Zitzmann et al.^16^ reported on 434 consecutive hypogonadal men also reported loss of erections around TT of 8 nmol/L, diabetes and depression at 10 nmol/L, obesity at 12 nmol/L and reduced vigour at 15 nmol/L. Hackett et al.^17^ demonstrated that in men with T2DM, symptoms improve in a similar fashion as testosterone levels improve with therapy.

The clinical response to T Therapy appears unrelated to the underlying aetiology,^18^ as recent trials have shown benefits in men without “classical HG,” as these text‐book conditions are relatively rare in the general population. It is a matter of clinical judgement and patient expectation as to whether the underlying conditions should be addressed first, but evidence suggests that better outcomes may be achieved if lifestyle, appropriate medications and T Therapy are combined.^19^

T Therapy is appropriate to treat ED, especially in men with TT levels below 8 nmol/L^7^, ^8^, ^9^ and to salvage ED treatment failures with oral medication especially at TT levels below 10.4 nmol/L.^20^ Appropriate interventions with testosterone supplementation reduce the needs for more invasive second‐ and third‐line treatment.^21^ These indications for T Therapy are often vitally important to the patient but considered to be of low importance by the physician not specialising in sexual dysfunction.

### 2.3 Testosterone therapy for men with testosterone deficiency is effective, rational, and evidence based

Several meta‐analyses of RCTs, notably by Corona et al.,^22^ have concluded that T Therapy in men with HG significantly improves sexual desire, erectile function (especially in men below 8 nmol/L), increases sexual activity, satisfaction and orgasm. In a meta‐analysis of 59 RCTs involving 5078 subjects, T Therapy was found not to significantly change body weight, body mass index (BMI) or waistline in RCTs but consistently improves lean muscle and decreases fat mass.^22^

In February 2016, the largest long‐term double‐blind placebo‐controlled study of T Therapy for HG, in 790 men over 65 years with 12‐month duration, was published.^23^ This showed significant improvements in sexual function and modest improvement in 6‐minute walking test, functional performance, mood, depression and fatigue. The composite benefits of these improvements are likely to translate into major quality of life and health economic benefit.^23^

Long‐term registry studies of over 10 years, however, have shown progressive weight loss and decreased waist circumference and BMI.^24^, ^25^ Cessation of therapy resulted in relapse and reversal of benefits within 6 months, meaning that patients were advised that therapy is likely to be lifelong.^20^, ^26^

Most RCTs of T Therapy were of relatively short duration, 6 months, or occasionally 12 months. T Therapy improved insulin resistance most marked in poorly controlled patients. There are small benefits in lipid metabolism but blood pressure is unaffected.^17^, ^27^ These benefits are more pronounced when combined with lifestyle modifications.^17^ T Therapy has consistently been shown to improve bone mineral density in the lumbar spine without significant improvement in hip scores.^28^ Lower urinary tract symptoms (LUTS) are improved by a mean of 2.2 points in RCTs,^27^ and depression scores are improved in uncontrolled studies.^29^ Most RCTs were conducted over a 3‐ to 12‐month duration and evidence strongly suggests that trials of T Therapy should be for a minimum of 6 months.^17^

Although HG is often associated with reduced fertility,^7^ T Therapy in younger men reduces LH and FSH levels and frequently causes infertility after 6‐12 months which is reversible in 60‐70% of men within 9‐12 months.^28^ Where fertility is an important issue for men with HG, then alternative therapy such as HCG or Clomiphene citrate (unlicensed in men) should be considered.^7^

### 2.4 There is no scientific basis for withholding T Therapy from men on the basis of age

Although it is commonly stated that testosterone declines with age, evidence from EMAS suggests that there is little decline in T levels between 40 and 75 in non‐obese men.^14^ In fact, over 80% of men will maintain normal T levels into old age, suggesting that the term “age‐related hypogonadism” is misleading. There is a fall in FT and bioavailable T secondary to the rise in SHBG with age, but primarily the increase in TD with age is related to increasing prevalence of obesity, T2DM, and chronic illness.^14^

Concerns over T Therapy in elderly men are based on the premature discontinuation of the Testosterone in Older Men trial 30. This involves 209 elderly frail men over 65 years randomised to receive either placebo or 100 g (twice the recommended dose at initiation of therapy) of topical testosterone gel, designed to assess frailty and muscle strength and not powered to detect major cardiovascular events (MACE), was terminated early as there were 23 cardiovascular‐related events (two deaths) in the 106 men in the testosterone group vs five in the placebo group, despite positive results in study end‐points. The study involved rapid escalation up to 150 mg per day, above the manufacturer's recommended dose, and many of the events were reported with inadequate validation.^31^

The clinical and physiological responses to T Therapy, especially increased muscle mass and strength, are seen in both younger and older men.^22^ These benefits may be of greater clinical and economic significance in older men, as reduced muscle mass and lower limb strength are strongly related to frailty and increased rate of falls.^32^

**Figure 2:**
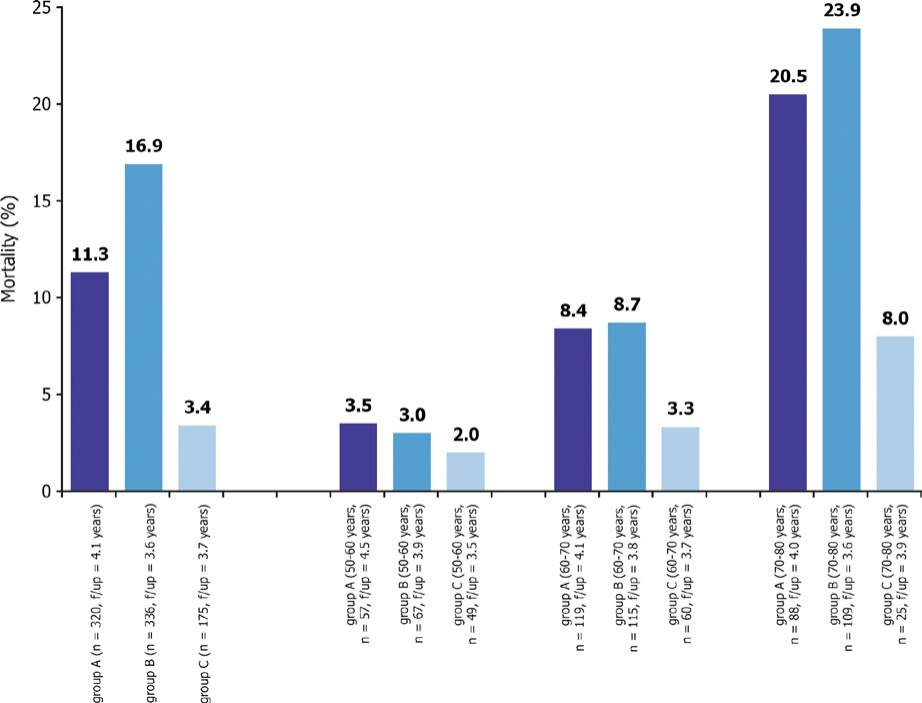
Trend for reduction in all‐cause mortality in T2DM appears greatest in men over 75^33^. Mortality in patients categorised by: Group A = normal testosterone; B= low testosterone untreated; C = low testosteron

Hackett et al. suggested a greater reduction in all‐cause mortality in men over 75^33^ (Figure 2). The traditional view that younger men would see greater benefit from improvement in sexual symptoms has not been supported by recent studies.^34^

### 2.5 Testosterone deficiency is associated with increased cardiovascular and all‐cause mortality

There is increasing evidence from multiple long‐term studies that HG is associated with increased cardiovascular and all‐cause mortality. A 10‐year study from Western Australia involving 3690 older men concluded that TT and FT levels in the normal range were associated with decreased all‐cause and cardiovascular mortality, for the first time suggesting that both low and high levels were associated with all‐cause mortality and higher levels of dihydrotestosterone (DHT)‐reduced cardiovascular risk.^35^ A recent Swedish study with a 14‐year follow‐up suggested a strong association between baseline testosterone and incident myocardial infarction (MI).^36^ Araujo et al. concluded that most studies involved issues in cohort selection and choice.^37^ They concluded that a decrease of 2.1 standard deviations in TT was associated with a 25% increase in mortality. Haring et al. looked at the data in terms of several statistical models and found that even after strict adjustment for comorbidities, there was a consistent link between mortality risk and testosterone level throughout the studies without proving causation^38^ (Table 3). Similar conclusions were drawn from meta‐analyses by Ruige et al.^39^ and most recently Corona et al.,^40^ where the focus was on cardiovascular disease as opposed to all‐cause mortality. All conclude that there is a consistent link between low testosterone and cardiovascular disease incidence and mortality, but this did not prove a pathogenic link, but Muraleedharan et al. concluded that low testosterone could be a “marker” of illness.^41^

**Table 3:** Association of low testosterone level with all‐cause mortality adjusted for multiple variables^33^

Cut‐off for the definition of low total testosterone (TT)	MMAS; 8 TT <6.94 nmol/L (200 ng/dL)	Wang: 34 TT <8 nmol/L (230 ng/dL)	Rancho Bernardo; 7 TT <8.36 nmol/L (241 ng/dL)	Male Veterana; 35 TT <8.7 nmol/L (250 ng/dL)	HIM; 36 TT <10.41 nmol/L (300 ng/dL)	EPIC; 6 TT <12.5 nmol/L (360 ng/dL)	Age‐specific cut‐off <10th percentile
Low TT (n)	34	69	82	98	241	474	
Model 1	1.59 (0.83;4.02)	1.96 (0.93;3.63)	2.21 (1.26;3.89)**	2.24 (1.41;3.57)**	1.33 (0.93;1.90)	1.28 (0.95;1.72)	2.21 (1.40;3.49)**
Model 2	2.12 (1.01;4.46)*	2.08 (1.12;3.86)*	2.33 (1.33;4,21)**	2.10 (1.34;3.29)**	1.28 (0.89;1.84)	1.20 (0.88;1.62)	2.26 (1.43;3.59)**
Model 3	2.50 (1.18;5.27)*	2.24 (1.21;4.17)*	2.53 (1.43;4.47)**	2.32 (1.38;3.89)**	1.37 (0.95;1.99)	1.28 (1.93;1.75)	2.35 (1.47;3.74)***
Model 4	2.68 (1.19;6.04)*	2.13 (1.06;4.26)*	2.56 (1.38;4.76)**	1.92 (1.18;3.14)**	1.11 (0.72;1.69)	1.10 (0.78;1.56)	2.25 (1.35;3.75)**

HR, hazard ratio; 95% CI, 95% confidence interval; CVD, cardiovascular disease; WC, waist circumference; DHEA, dehydroepiandrosterone sulphate. Model 1: adjusted for age. Model 2: adjusted for model 2, smoking (three categories), high‐risk alcohol use and physical activity. Model 4: adjusted for model 3, renal insufficiency and DHEAS. *P=.05; **P=.01; ***P=.001.

Six published studies generally involving small samples have shown that low TT and FT are associated with coronary artery disease (CAD), and four have shown no association.^42^ Four studies have shown inverse associations between low TT and FT (Table 3) and the severity of CAD. One involved 803 men assessed by Gensini score, based on the location and number of stenotic coronary artery segments and degree of luminal narrowing. Once again, such studies do not establish whether low TT or FT is a cause or a consequence of CAD.^42^ The vascular role of testosterone was recently reviewed by Kelly and Jones.^43^ The evidence that testosterone replacement in four retrospective studies improves survival does suggest that testosterone may have a beneficial effect.^44^, ^45^, ^46^, ^47^

### 2.6 The evidence does not support an increased cardiovascular risk associated with T Therapy

Possible mechanisms for adverse cardiovascular disease events with T Therapy may arise through a 6% increased rate of polycythaemia, related to multiple mechanisms.^48^ By conversion to oestradiol, there is direct stimulation of erythropoiesis in the bone marrow. Testosterone also stimulates erythropoietin synthesis in the kidney and in turn increases erythropoiesis and T‐induced increase of hepatic transcription factors with decreased hepcidin and as a consequence of increased iron usage.^49^ It has been suggested that supra‐physiological levels associated with short‐acting injections may exaggerate this effect^27^ as may the conversion of gels to DHT by the action of 5‐alpha reductase in the skin.^28^ A full list of adverse events associated with T Therapy is shown in Table 4. These include polycythaemia with increase of haematocrit and haemoglobin, gynaecomastia, loss of head hair, acne and other skin disorders, increased aggressiveness and hyper sexuality.

**Table 4:** Adverse effects of testosterone therapy

Formulation	Adverse effects
Injections i.m.	Pain at injection site
	Fluctuations in mood, energy and sexual desire
	Coughing immediately after injection (POME)
Transdermal gels	Potential risk of gel transfer to others in close contact
	Skin irritation
	Fluctuations in absorption
Subcutaneous pellets	Frequent skin reactions at the application site
Oral 17‐α‐alkylated	Hepatotoxicity
	Cholestasis
	Peliosis hepatis
	Hepatic tumour
	Marked decrease in HDL cholesterol

Other side effects include polycythaemia with increase of haematocrit and haemoglobin, gynaecomastia, loss of head hair, acne and other skin reactions, increase of aggressiveness and hyper sexuality.

Increased levels of DHT may be associated with associated increased thrombotic risk and fluid retention provoking hypertension and heart failure.^4^ Studies with high‐dose testosterone use in body builders have shown reductions in high‐density lipoprotein cholesterol,^45^ whereas randomised studies of appropriate dose T Therapy have shown minimal adverse effect of T Therapy or even improvement.^24^, ^25^

A retrospective US study of 8709 men^2^ with baseline TT of 10.4 nmol/L or less undergoing coronary angiography involved follow‐up for a mean of 840 days. In the cohort of 7486 patients not receiving T Therapy, 681 died, 420 had MIs and 486 had strokes. Among 1223 patients receiving T Therapy, 67 died, 23 had MIs and 33 had strokes. At first sight, these results would closely agree with the findings of other studies, but a complex statistical analysis (using >50 covariates) reversed the trend and concluded that there was a greater risk in the T Therapy group. There were concerns that 1132 patients experiencing events were excluded because they were prescribed T Therapy after the event when surely these should have been included in the untreated group, increasing the events by 70%. Furthermore, there were no data on whether there was a correct diagnosis of TDS before T Therapy, none on compliance and some patients did not continue T Therapy, ‘and mean TT levels on T Therapy were at lower end of normal suggesting many were under treated. When challenged, the authors revised the number to 132, but conceded that 104 women had wrongly been included in the results.

Finkle et al.^3^ studied prescribing data in men treated with T Therapy, but with no data on blood results or symptoms. Non‐fatal coronary events were the major end‐point, assessed in the 12 months before and 3 months after therapy, even though benefits of T Therapy would take much longer and other studies had excluded the first 3 months from analysis as the events would be likely to be related to the pre‐existing condition. Most importantly, fatal cardiovascular events and all‐cause mortality data were not collected despite the major impact of T Therapy in other studies being seen on mortality and not event numbers. Twelve‐month posttreatment data were collected but not presented. The event rates within the groups prior to treatment were strangely identical. They reported a small increase in non‐fatal cardiac events in men commenced on T Therapy, more marked in those with increased risk. Overall events in the study were lower than predicted from comparable research. They failed to report deaths, failing to realise that a treatment that reduced mortality was likely to increase non‐fatal events. The design was not prospective, casting doubts on the validity of retrospective assessment for the pretreatment 12‐month period. Although widely quoted in public media, several design flaws and statistical analyses have discredited this article. A meta‐analysis of 27 placebo‐controlled trials of T Therapy lasting more than 12 weeks by Xu et al.^50^ concluded that T Therapy may increase the risk of cardiovascular‐related events, whereas other meta‐analyses did not, but most studies involved small cohorts with a small number of events. A further meta‐analysis by Corona et al.^51^ concluded that T Therapy was not associated with increased risk, and in certain cohorts, there was evidence of reduced events. They were critical of Xu et al. for their selection and inclusion of studies. Findings from the Basaria et al. paper heavily skewed the Xu findings, and the inclusion of a Scandinavian study involving an unlicensed oral formulation in men with advanced liver cirrhosis suggested selection bias.^51^

A prospective study of 587 men with T2DM^44^ involved 5.8‐year follow‐up. Low testosterone was defined as TT <10.4 nmol/L. Fifty‐eight men were treated with testosterone for 2 years or more. The mortality rate was 20% in the untreated group and 9.1% in the normal group independent of comorbidities and therapies. Mortality was 8.6% in the treated group. A similar retrospective US study involved 1031 hypogonadal men, with 372 on T Therapy.^45^ The cumulative mortality was 21% in the untreated group vs 10% in the treated group, with the greatest effect in younger men and those with T2DM. Both articles were criticised for possible selection bias, but the strengths included reliable pretreatment diagnosis and accurate reporting of medications. Hackett et al.^33^ followed up 857 men with T2DM for 4 years following baseline testosterone measurement. Patients had been randomised to long‐acting testosterone undecanoate (TU) or placebo assessment during a randomised controlled study. The investigators confirmed that low baseline TT and FT were associated with increased all‐cause mortality over a 4‐year follow‐up. They reported that T Therapy and the use of phosphodiesterase 5 inhibitors (PDE5Is) were independently associated with reduced all‐cause mortality, with the greatest benefit from both T Therapy and PDE5Is being seen in older men.

Baillargeon et al.^52^ compared acute myocardial infarction rates for 6355 men over 8 years, receiving at least one testosterone injection compared with a matched placebo group, and found no overall increase in events. In the quartile at greatest risk, there was a significant reduction in events and mortality. The authors found no increased risk from venous thromboembolism. The same authors have shown significant reductions in acute hospital admission in men treated with T Therapy compared with an untreated cohort.^52^

Anderson et al.^53^ searched electronic medical records between 1996 and 2011 to identify 5695 men who had a low initial TT level, a subsequent testosterone level, and >3‐year follow‐up. Levels were correlated with testosterone supplement use. Primary outcomes were a composite of death, non‐fatal MI, and stroke, MACE and death alone. T Therapy in men with low testosterone was associated with reduced MACE and death over 3 years compared with no or ineffective supplementation. This study suggested that the favourable impact of T Therapy was predominantly on mortality, rather than number of events, and benefits were associated with achieving therapeutic levels of testosterone, with no suggestion of increased risk with sustained higher serum levels. The same group^53^ have shown significant reduction in cardiovascular events in a cohort of hypogonadal men with angiographically diagnosed CAD.

**Figure 3:**
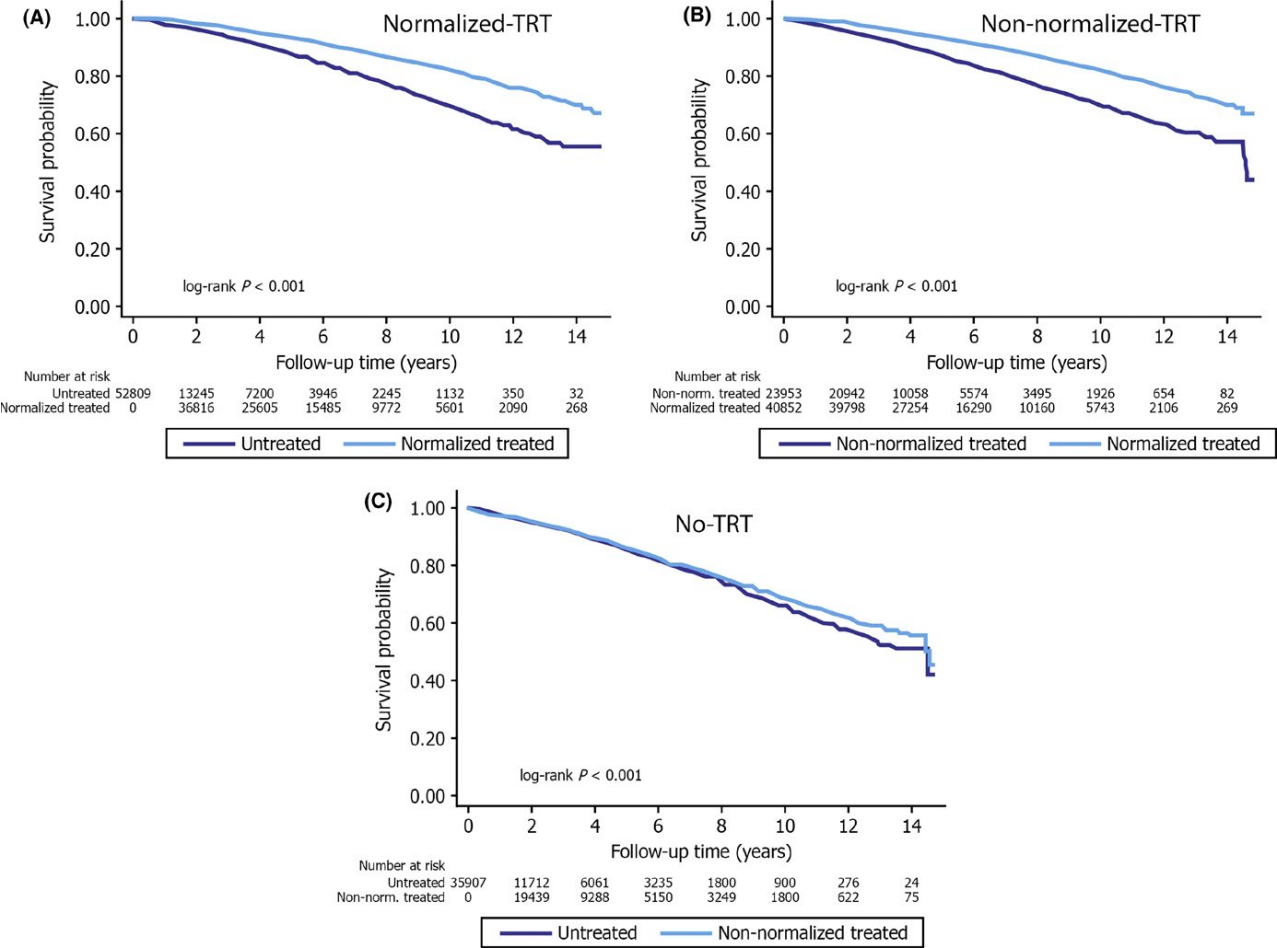
Kaplan‐Meier curve depicting the all‐cause mortality among different propensity‐matched study groups^46^

Sharma et al.^46^ retrospectively evaluated 83,010 male veterans with documented low TT levels. The subjects were categorised into three groups: T Therapy with resulting normalisation of TT levels (group 1); T Therapy without normalisation of TT levels (group 2); and did not receive T Therapy (group 3). The all‐cause mortality (HR 0.53, 95% CI 0.50‐0.55), risk of MI (HR 0.82, 95% CI 0.71‐0.95) and stroke (HR 0.70, 95% CI 0.51‐0.96) were significantly lower in group 1 vs group 2 (n=25 701, median age 66 years, mean follow‐up 4.6 years) (Figure 3).

These studies present the most compelling evidence to date for the safety of T Therapy in patients with reduction in mortality clearly defined HG treated to the therapeutic range, suggesting that studies with negative outcomes usually included inadequate diagnosis and little evidence of effective therapeutic levels or adequate follow‐up (Figure 3). Several registry studies have published data with over 7‐year follow‐up, with no suggestion of increased mortality.

Recent evidence suggests that responsiveness to T Therapy is dependent not only on the serum testosterone concentration but also on the length of CAG repeats on the androgen receptor. Longer CAG repeats are associated with more severe symptoms and reduced response to therapy at standard doses. The large ethnic variations in CAG repeats within different ethnic groups are of particularly important in the UK population.^54^, ^55^

### 2.7 There is no evidence that supports any increase in the risk of cancer of the prostate with testosterone replacement therapy

Guidelines of the ES, ISSM, EAU, ESSM, ISSM and BSSM all conclude that there is no evidence that T Therapy is associated with increased risk of prostate cancer.^7^, ^8^, ^9^, ^10^, ^11^ Recent studies suggest that lower levels of testosterone are associated with the risk of poorly differentiated cancers and greater risk of positive biopsy.^56^

There is now strong evidence linking low T concentrations to aggressive, high‐grade prostate cancer, higher rates of positive biopsy, biochemical recurrence and disease progression in men involved in active surveillance.^57^

The 2015 EAU guidelines 7 make the following statement.

T Therapy results in a marginal increase in PSA and prostate volume, plateauing at 12 months. Previous fears that T Therapy might increase the risk of prostate cancer have been contradicted by a number of meta‐analyses.

There are insufficient long‐term data available to conclude that there is safety from prostate cancer with T Therapy. Prostate monitoring, therefore, remains indicated. Subjects with a substantial or continuous increase in PSA level (taking the level 6 months^7^ after treatment initiation as baseline) need to be investigated to exclude prostate cancer.

### 2.8 A major research initiative to explore the benefits of T Therapy in cardio‐metabolic disease is overdue

Most reviews conclude that a long‐term RCT is required to definitively answer the complex issues around T Therapy.

The Testosterone for the prevention of Diabetes Mellitus (T4DM) (t4dm.org.au) study in Australia^58^ involves younger (n=1500), obese men with glucose intolerance and testosterone levels of 8‐11 nmol/L, randomised to long‐acting TU or placebo, to establish whether T Therapy will reduce the development of T2DM. This might provide important answers for younger men, but will not answer questions about risk/benefits in older populations.

It is unlikely that definitive answers will be found to the many questions raised in this article without huge independent funding. With considerable evidence of benefit associated with T Therapy for HG, it is unlikely that ethical approval will be granted for sufficiently powered placebo‐controlled studies lasting several years.

## 3 Conclusions

Testosterone deficiency is a well‐established, significant medical condition with defined clinical symptoms and is associated with increased cardiovascular and all‐cause mortality. Treatment is effective, evidence based and safe. Recent studies suggest that T Therapy resulting in sustained normalisation of serum levels is probably associated with reduced mortality. Currently available T Therapy treatment modalities and their advantages and disadvantages are outlined in Table 5. T Therapy is associated with multiple benefits maybe highly relevant to the patient but underestimated by specialist physicians focused on specific outcomes. Until the definitive well‐powered long‐term study is published, we hope that these consensus statements will enable patients to be treated on best available evidence (Table 6).

**Table 5:** TRT treatment options^7^

Route of administration	TRT formulation	Advantages	Disadvantages
Transdermal gel	Transdermal testosterone	Quick onset Steady‐state testosterone levels	Skin irritation at the site of application Risk of interpersonal transfer Long‐term patient non‐compliance
Oral	Testosterone undecanoate	Absorbed through the lymphatic system (reduction of liver involvement)	Variable levels of testosterone Several doses needed per day with intake of fatty food
Intramuscular	Testosterone undecanoate	Steady‐state testosterone levels without fluctuation Three monthly injections improving patient compliance	Long‐acting preparation—does not allow drug withdrawal in case of side effects
	Testosterone enanthate/propionate injections	Short‐acting preparation that allows drug withdrawal in case of onset of side effects	Short‐term—One injection every 2‐3 weeks Fluctuation of testosterone levels

**Table 6:** BSSM policy statements on testosterone deficiency

BSSM policy statements on testosterone deficiency
1. Testosterone deficiency is a well‐established, significant medical condition
2. Testosterone deficiency has well‐established symptoms
3. Testosterone therapy for men with testosterone deficiency is effective, rational and evidence based
4. There is no scientific basis for withholding testosterone therapy from men on the basis of age
5. Testosterone deficiency is associated with increased cardiovascular and all‐cause mortality
6. The evidence does not support an increased cardiovascular risk associated with testosterone therapy
7. There is no evidence that supports any increase in the risk of cancer of the prostate with testosterone replacement therapy
8. A major research initiative to explore the benefits of testosterone therapy in cardiometabolic disease is overdue

## Author contributions

All authors reviewed and edited the publication, which was produced by Geoff Hackett.

## Disclosures

Geoff Hackett is an occasional speaker for Bayer, Besins and Menarini Asif Muneer is an occasional speaker for Bayer and Eli Lilly.

Mike Kirby has received funding for research, conference attendance, lecturing and advice from the pharmaceutical industry including Astellas, Pfizer, Takeda, Bayer, MSD, BI, Lilly, GSK, AZ and Menarini.

He is also the editor of PCCJ and is on several NHS advisory boards including the Prostate cancer Risk Management Programme and the Prostate Cancer advisory Group

T. Hugh Jones has received research grants from Bayer, Besins Healthcare, ProStrakan. Consultancy from Clarus Therapeutics, Mereo Biopharma; as well as Honoraria for educational lectures from Bayer, Besins Healthcare, ProStrakan. Advisory Boards for Bayer, Besins Healthcare, Lilly, Merck. David Edwards has received support for accommodation, travel and honoraria for presenting from several Pharma companies including Bayer.
